# Verrucous Carcinoma of the Vulva: A Case Report and Review of the Literature

**DOI:** 10.3389/fsurg.2016.00008

**Published:** 2016-02-11

**Authors:** Jean Bouquet de Jolinière, F. Khomsi, J. M. Gothuey, L. Guillou, A. Fadhlaoui, J. B. Dubuisson, A. Feki

**Affiliations:** ^1^Department of Gynecologic and Oncologic Surgery, HFR Cantonal Hospital of Fribourg, Fribourg, Switzerland; ^2^Argotlab, Department of Pathology, Lausanne, Switzerland

**Keywords:** vulva carcinoma, verrucous carcinoma, vulva surgery, HPV, condyloma

## Abstract

Verrucous carcinoma of the vulva is a rare lesion (1). Affecting essentially postmenopausal women, this lesion is a distinct and particular entity in vulval carcinoma classification and its scalability is uncertain and unpredictable. Here, we present a case concerning a 48-year-old patient, without follow-up after a condyloma acuminate of the vulva (large left lip). The origin of this case will be discussed in this article. The treatment decided was only surgical. A review of literature shows the rarity of this lesion of the female genital tract.

## Introduction

The verrucous carcinoma is an uncommon lesion in the vulvar cancer classification. Effectively, its characterization is defined by a slow growing, no metastasis or lymph node involvement (2), and its appearance is exophitic (3) and impressive. We observe very often a destructive alteration of the tissues. Verrucous carcinoma is a tumor with thickness that can invade and compress the underlying stroma with “pushing margins” (1, 2). The induration resulting from the surrounding tissue and enlargement of regional lymph nodes may mislead the clinician into making an erroneous diagnosis of squamous cell carcinoma (1, 2). A biopsy is absolutely necessary before any decision and treatment.

When the incidence of malignancy is low (1–2% of genital cancers), the treatment is essentially surgical, with a large excision and a strict follow-up.

It is essential to analyze the pathogenesis of this lesion: recently, the role of HPV (human papilloma virus) has been indicated (3, 4). But many others factors can create a “fertile ground” for the development of the lesion: smoking, hormonal deficiency, and diabetes, each of them associated is with an HPV infection, but the majority describes lesions without HPV infection (1, 5).

In literature (1, 6, 7), most surgeons recommend only a local excision as the best treatment without any lymph nodes removal. Other treatments are inefficient (radiotherapy, local chemotherapy, cryotherapy, etc.). In these cases, the recurrence rate is high. Because recurrence may occur if surgical resection margins are involved by the tumor, the pathologist should carefully consider these margins: it is the reason why a large and deep surgery is necessary. It is important to note that the recurrence of verrucous carcinoma announces a bad prognosis (6).

Otherwise, others localizations may be found in the anal channel, vagin (6, 8).The partner must be examined systematically. The epidemiology, pathogenesis, and localization are those of HPV.

## Clinical History

This 48-year-old patient consulted 2 years before for a pruritus on the left lip. The vulvoscopy showed a condyloma acuminate; the gynecologist had not performed a biopsy and given a cream with podophyllin.

After the failure of treatment, another gynecologist decided to vaporize the lesion with a CO_2_ laser, without a previous biopsy. The patient developed in 2 years (without following) a giant tumor (2 cm of diameter minimum).

A first resection by the third gynecologist is performed, with partial removal of the clitoris.

The results confirm a verrucous carcinoma (1.8 × 1.2 × 0.3), the margins are in the tumor (left side and deep section) (dermal invasion of 1.9 mm).

**Figure 1:**
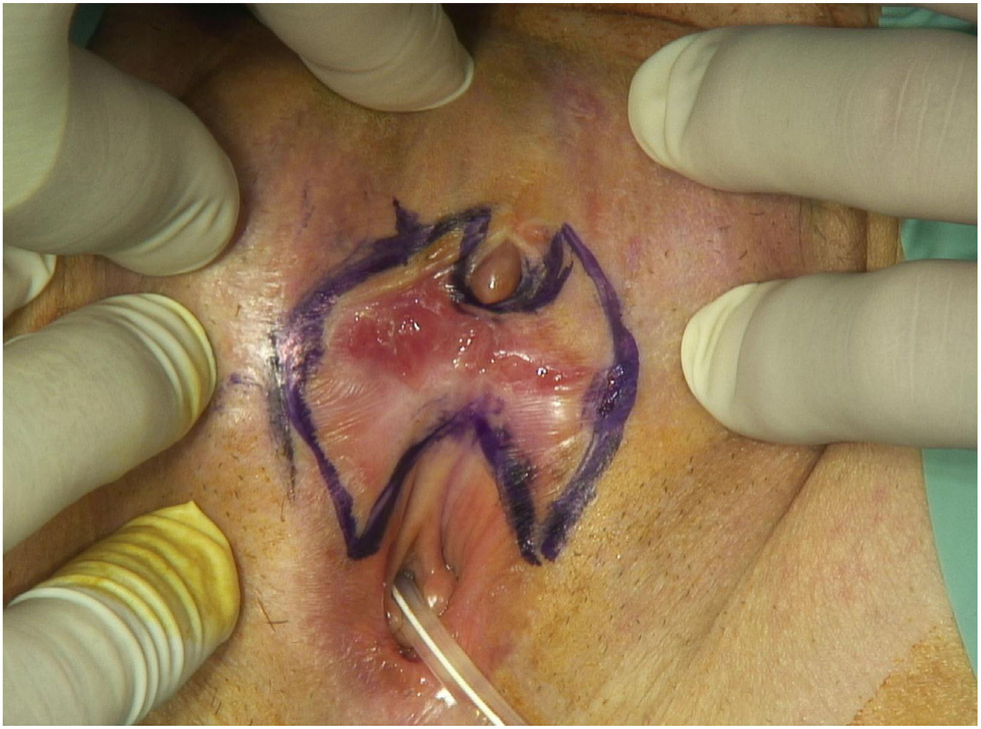
**Drawing in blue for surgical incision**.

**Figure 2:**
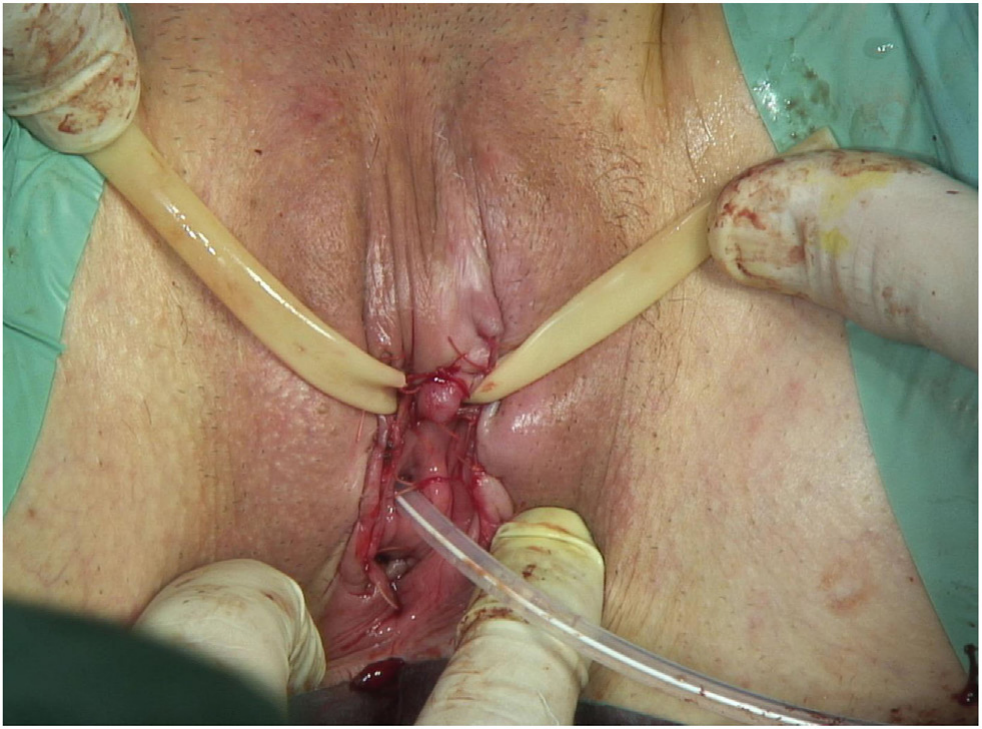
**End of surgery**.

The decision of multidisciplinary meeting is for a partial vulvectomy in our department with a vulvoplasty and a replantation of the clitoris (Figures 1 and 2).

**Figure 3:**
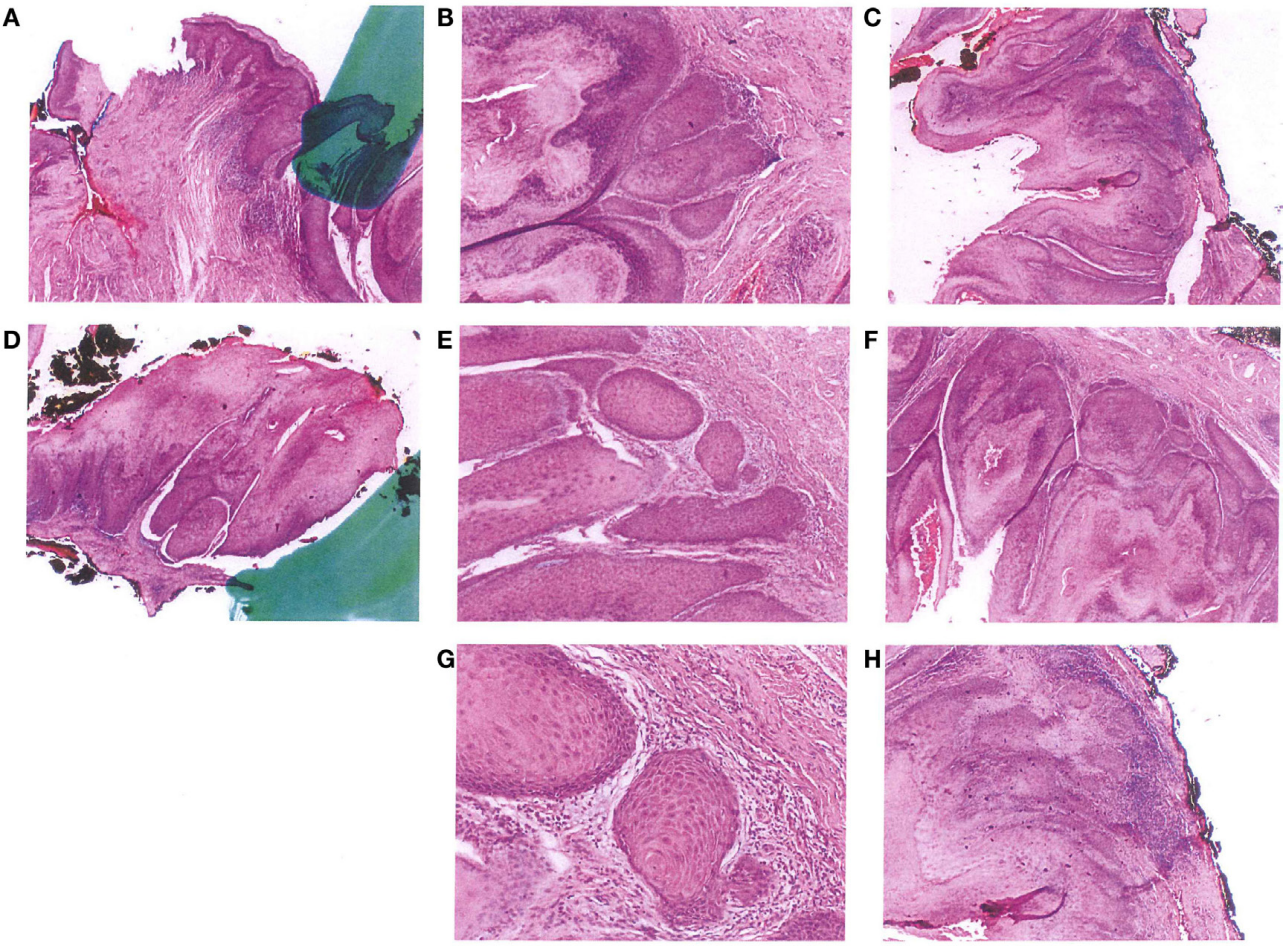
**Pathological pictures**.

The final pathology shows total free margins (Figures 3A–H). The anatomical and functional results are good. No complementary treatment was decided. But follow-up was organized. The patient must be seen every 6 months. The PAP is normal and the partner safe.

## Discussion

The verrucous carcinoma is a variant of squamous carcinoma (<1% of vulvar cancer).

The main reasons for the patient consulting are itching and pain (vulvodynia).

The pathological report must include histological type, differentiation, and infiltration: measurement of tumor penetration from the dermis papillary to the deep edge of the tumor invasion with minimum margins in millimeter.

Many authors have found HPV genome in the carcinoma tissue (9).

It is always necessary to evaluate the cervix (PAP, colposcopy) because vulva and cervix have a common embryologic origin (squamous tissue) from ectodermic differentiation. In some cases, a cervix dysplasia (CIN) may be found associated with HPV disease and must be treated.

In our case, the pathologic description shows in the vulvectomy: a squamous epithelium, with a mature orthokeratosic and kyperkerathosis surface without koilocytes, with sparse lymphocytic infiltrate. All surgical margins are in healthy tissue.

In Figures 1 and 2, the surgical incision was large. Indeed, the surgery must avoid a potential invasion of deep adjacent structures. A strict follow-up is performed to research any recurrence.

Finally, the prognosis is, anyway, good, depending of an initial extensive surgery.

## Ethics Statement

Written informed consent was obtained from the patient prior to presenting the case.

## Author Contributions

Jean Bouquet de Jolinière, F. Khomsi, J. M. Gothuey, L. Guillou, A. Fadhlaoui, J. B. Dubuisson and A. Feki: All have participated in this work.

## Conflict of Interest Statement

The authors declare that the research was conducted in the absence of any commercial or financial relationships that could be construed as a potential conflict of interest.
